# Periphyton Function in Lake Ecosystems

**DOI:** 10.1100/tsw.2002.294

**Published:** 2002-05-29

**Authors:** Yvonne Vadeboncoeur, Alan D. Steinman

**Affiliations:** Department of Biology, McGill University, 1205 Avenue Docteur Penfield, Montreal, Quebec H3A 1B1, Canada

**Keywords:** periphyton, lakes, nutrient cycling, primary productivity, periphytonphytoplankton interactions, benthic-pelagic coupling, aquatic food webs

## Abstract

Periphyton communities have received relatively little attention in lake ecosystems. However, evidence is increasing that they play a key role in primary productivity, nutrient cycling, and food web interactions. This review summarizes those findings and places them in a conceptual framework to evaluate the functional importance of periphyton in lakes. The role of periphyton is conceptualized based on a spatial hierarchy. At the coarsest scale, landscape properties such as lake morphometry, influence the amount of available habitat for periphyton growth. Watershed-related properties, such as loading of dissolved organic matter, nutrients, and sediments influence light availability and hence periphyton productivity. At the finer scale of within the lake, both habitat availability and habitat type affect periphyton growth and abundance. In addition, periphyton and phytoplankton compete for available resources at the within-lake scale. Our review indicates that periphyton plays an important functional role in lake nutrient cycles and food webs, especially under such conditions as relatively shallow depths, nutrient-poor conditions, or high water-column transparency. We recommend more studies assessing periphyton function across a spectrum of lake morphometry and trophic conditions.

## INTRODUCTION

Primary producers form the energetic base of most ecosystems on this planet. In lentic systems, algae are most often the dominant primary producers, and are responsible for carbon fixation and sequestration of essential nutrients such as nitrogen and phosphorus, which then become available to consumers. As such, algae are critical ecosystem components of both nutrient cycles and food webs in lakes. Early limnologists had a strong sense of the importance of benthic primary producers in lake ecosystem function[1,2]. However, in the last several decades both empirical and experimental limnology have had an overwhelmingly pelagic focus[3,4], with little quantitative integration of benthic processes into whole-lake dynamics. Exceptions to this generalization include the in-depth study on the roles of macrophytes in European shallow lakes[5,6] and the recent focus on benthic-pelagic coupling[7]. Lowe[8], in a recent review of periphyton patterns in lakes, noted that our knowledge of basic functional attributes of lake periphyton is still rudimentary. In this paper, we explicitly address the function of periphyton in lake ecosystems. We first address the question of the relative importance of periphyton communities to total lake primary production and then focus on two broad features regarding the role periphyton play in nutrient and carbon dynamics at the whole-lake scale: (1) interactions between periphyton and other primary producers that affect ecosystem nutrient cycling and (2) the importance of periphyton as an energy base for food webs.

### Periphyton Contributions to Whole-Lake Primary Production

Before assessing the functional role of periphyton in lakes it is useful to evaluate total rates of production by benthic algae at the whole-lake scale and to compare them with rates of phytoplankton production. Table 1 is a compilation of literature studies for which total production of phytoplankton, periphyton, and macrophytes was estimated at the whole-lake level. We restricted our presentation to studies that measured periphyton production or biomass on natural substrata because, as discussed below, substratum has strong effects on periphyton primary production and biomass. Table 1 is striking in that there are so few studies for which estimates of primary production among all autotrophs within lakes have been attempted. Possible reasons for the paucity of comparisons include: (1) limnologists perceive littoral and pelagic habitats to be discrete and noninteracting; (2) the functional importance of periphyton in lake ecosystems is assumed to be minimal; or (3) measuring periphyton productivity is logistically difficult compared with phytoplankton. However, it is clear that productivity of periphyton can be similar to or exceed phytoplankton, especially at low and intermediate levels of phytoplankton production, indicating that periphyton can be important in lake ecosystems processes.

We currently lack a formal conceptual structure within which to examine the role of periphyton in lake nutrient cycles and food webs. Studies of phytoplankton are rarely presented outside of the context of lake trophic status, and there are well-developed empirical relationships between phytoplankton and nutrient concentrations[9,10,11,12], grazer densities[13], and morphometry[14,15,16]. Unfortunately, there are no similar relationships developed for lake periphyton (but see Dodds et al.[17] for streams), so it is difficult to make predictions about how periphyton contributions to whole-lake primary production vary across environmental gradients. Conceptual models for patterns in periphyton production with lake trophic status have been proposed[7,18,19]. These models postulate that periphyton has first access to sediment nutrients but the communities are increasingly shaded by phytoplankton along eutrophication gradients. The few tests of these models have generally supported them[7,20,21]. However, the high spatial variability in periphyton community structure and production[22] has frustrated detection, and perhaps the search for overall patterns among lakes. Instead, much effort has been spent on describing this spatial heterogeneity on a lake-by-lake basis. Here we propose a hierarchical framework within which periphyton function (e.g., metabolism, nutrient cycling, energy flow) in lakes can be explored (Fig. 1). At the coarsest level, landscape dynamics will influence lake morphometry, which in turn determines overall habitat availability

**TABLE 1**
**Total Annual Whole-Lake Primary Productivity Expressed per Square Meter of Lake Surface Area**

| Lake | Location | Mean Depth (m) | Surface Area (ha) | Trophic Status | Annual Primary Production ($g\ C\ m^{-2}\ year^{-1}$) Phyto-Plankton | Peri-phyton | Macro-phyte | Periphyton ALGAL (% total productivity) | Source |
|---|---|---|---|---|---|---|---|---|---|
| Tundra Ponds[a] | Alaska, USA | 0.2 | <1 | Oligotrophic | 0.7 | 8.4 | 0 | 92.3 | 138 |
| Ikroavik | Alaska, USA | 2.0 | 1200 | Oligotrophic | 2.2 | 2.3 | 0 | 51.1 | 138 |
| Lake 18 | North West Territories, Canada | 5.2 | 282.8 | Oligotrophic | 5.2 | 33.7 | - | 87 | 139 |
| Borax | Michigan, USA | 0.7 | 43 | Oligotrophic | 93 | 70 | 1.2 | 42.9 | 140 |
| Wingra | Wisconsin, USA | 2.0 | 139.6 | Eutrophic | 438 | 3.1 | 117 | 0.7 | 141, 142 |
| Marion | British Columbia, Canada | 2.2 | 13.3 | Oligotrophic | 8 | 31 | 18 | 79.5 | 143, 144,145 |
| Paul[b] | Michigan, USA | 3.7 | 1.7 | Oligotrophic | 42 | 139 | 0 | 76.8 | 21 |
| West[b] | Michigan, USA | 4.7 | 3.4 | Oligotrophic | 40 | 154 | 0 | 79.4 | 21 |
| East[b] | Michigan, USA | 4.9 | 2.3 | Oligotrophic | 62 | 64 | 0 | 50.8 | 21 |
| Peter[b] | Michigan, USA | 5.7 | 2.7 | Oligotrophic | 77 | 150 | 0 | 66.1 | 21 |
| Kalgaard | Denmark | 4.7 | 10.5 | Oligotrophic | 24.1 | 0.5 | 21.5 | 2.0 | 146 |
| Mirror | New Hampshire, USA | 5.8 | 15 | Oligotrophic | 29 | 2.1 | 1.4 | 6.8 | 147 |
| Lawrence | Michigan, USA | 5.9 | 5 | Oligotrophic | 43 | 40 | 88 | 48.2 | 148 |
| Eagle | California, USA | 7.0 | 12150 | Eutrophic | 117 | 14.2 | 0 | 10.8 | 149 |
| Lake 240[c] | Ontario, Canada | 6.1 | 441000 | Oligotrophic | 50 | 0.9 | 0.9 | 1.8 | 150 |
| Lake 239[c] | Ontario, Canada | 10.5 | 561000 | Oligotrophic | 82 | 0.81 | 0.81 | 1.0 | 150 |
| Char | North West Territories, Canada | 10.2 | 52.6 | Oligotrophic | 2.22 | 8.44 | 8.44 | 79.2 | 127 |
| Myvatn | Iceland | 2.3 | 2910 | Eutrophic | 60 | 270 | 50 | 81.8 | 29 |
| Esrom | Denmark | 13.5 | 1730 | Eutrophic | 170 | 35 | 19 | 17.1 | 29 |
| Thingvallavatn | Iceland | 34.1 | 8300 | Oligotrophic | 67.5 | 29 | 10 | 30.1 | 29 |
| Batorin[d] | Byelorussia | 3.0 | 630 | Eutrophic | 177 | 4.3 | 3.6 | 2.4 | 151 |
| Kiev[d] | Ukraine | 4.0 | 99000 | Eutrophic | 81 | 97 | 6.4 | 54.5 | 151 |
| Myastro[d] | Byelorussia | 5.4 | 1300 | Eutrophic | 158 | 10 | 6.2 | 6.0 | 151 |
| Krasnoye[d] | Russia | 7.0 | 900 | Mesotrophic | 106 | 14 | 16 | 11.7 | 151 |
| Mikolasjskie[d] | Poland | 11 | 500 | Eutrophic | 226 | 18.7 | 10.8 | 7.6 | 151 |
| Naroch[d] | Byelorussia | 11 | 8000 | Mesotrphic | 48 | 50 | 5.9 | 51.0 | 151 |
| Paajarvi[d] | Finland | 14 | 1300 | Oligotrophic | 26 | 1.8 | 0.9 | 6.5 | 151 |

*Note:* The macrophyte category includes only submerged and floating leaved macrophytes, not emergent taxa. The % periphyton category represents the contribution of periphyton to total algal productivity (phytoplankton + periphyton) and does not include macrophyte production. [a.] Primary production values are averaged for six small adjacent tundra ponds. [b.] Annual rates of production were estimated from hourly rates by assuming a 5-month growing season and an average day length of 14.5 h. [c.] The values for benthic primary production are probably underestimates because productivity was measured only on rocks, not on the extensive areas of unconsolidated sediments. Epipelic algae often have higher rates of production than epilithon (see text). [d.] Productivity was converted from MJ to g C assuming that 1 MJ = 26 g C.

for periphyton relative to phytoplankton. Within a lake, habitat heterogeneity and substratum composition in the littoral zone will strongly influence the abundance, taxonomic structure, and productivity of periphyton. Finally, phytoplankton-periphyton interactions will modify the role of periphyton in lake ecosystems across gradients of phytoplankton productivity.

## *Effects of Morphometry*

The influence of periphyton at the landscale scale is mediated by lake size and shape. Across a gradient from small ponds to large deep lakes, morphometry determines the relative amount of benthic and pelagic habitat available to primary producers[14]. Benthic substrata only constitute available habitat if there is sufficient light to allow periphyton photosynthesis. Water attenuates light, ultimately limiting the depth to which periphyton can grow. Additionally, there is an inverse

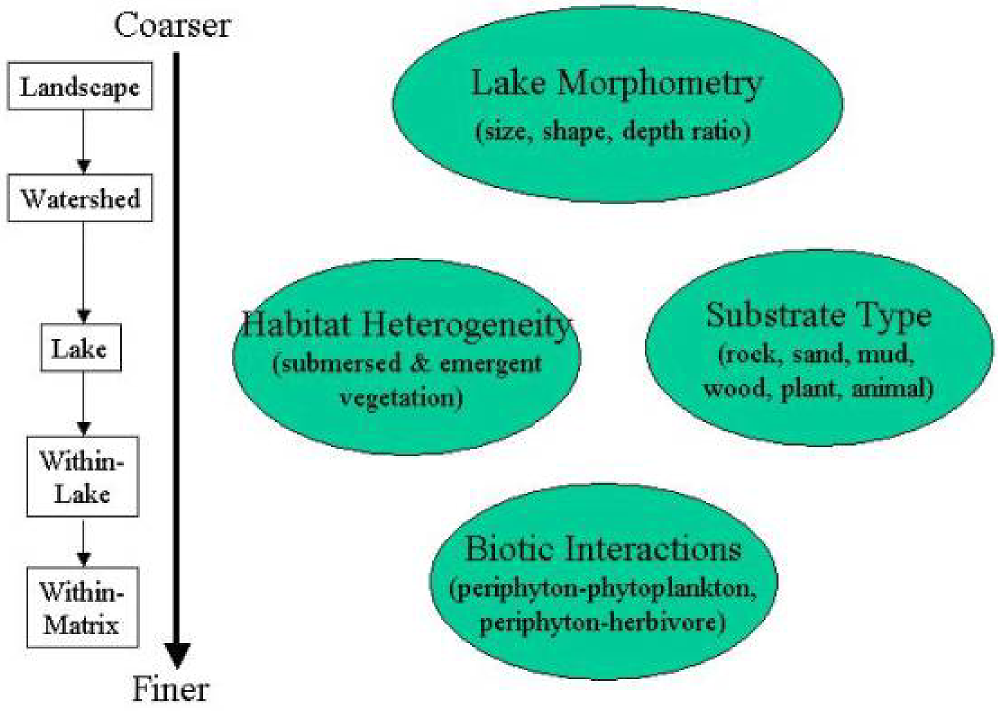

**FIGURE 1.** Hierarchical arrangement of possible factors influencing periphyton function in lake ecosystems, ordered from the coarsest level (top) to finest level (bottom). See text for additional details.

relationship between mean depth and the ratio of periphyton habitat (sediment surface area) to phytoplankton habitat (epilimnetic volume)[15]. Both of these simple morphometric relationships will cause the potential contribution of benthic algae to whole-lake primary production to decline as a function of depth[3]. For example, only a narrow band of littoral zone receives adequate light in large deep lakes such as Lake Tahoe, California[23], and Lake Taupo, New Zealand[24]. Each of these lakes has well-developed, productive littoral epilithic mats that extend >30 m and constitute 60 to 90% of littoral zone primary production, but account for only 2 to 5% of whole-lake primary production. However, the relationship with depth is extremely variable because shallow lakes can be clear and dominated by periphyton, but they also can have limited transparency because of phytoplankton[25,26], suspended solids[27], or high concentrations of dissolved organic matter (DOM). There is also an inverse relationship between lake depth and phytoplankton concentration. Thus, shallow lakes will tend to have higher phytoplankton concentrations (and hence higher light attenuation coefficients) than deep lakes with similar nutrient concentrations[15,16]. Although habitat availability puts an upper limit on the relative contribution of periphyton to whole-lake primary production in large, deep lakes, this contribution can still be substantial. For instance, Lake Thingvallavatn in Iceland has a mean depth of 34 m, and benthic algae compose 30% of whole-lake primary production. Figs. 2 and 3 show hypothetical examples of the relationship between relative contribution of benthic primary production vs. depth ratio and water color, respectively. Conceptually, these relationships are shown as biphasic, with benthic production accounting for approximately 50% of total primary production in lakes with very low depth ratios (i.e., extensive shallow areas; see below for definition of depth ratio), and then leveling off at between 5 and 10% of total production as the depth ratio increases. Data from a variety of lakes are needed to define the shape of the curve (linear, curvilinear, etc.) or identify the percent of benthic production where a break-point occurs. DOM has negative effects on phytoplankton[28], but we expect the impact may be greater on periphyton because periphyton does not circulate in the water column and is exposed to a relatively narrow range of light intensities. The relative contribution of benthic production is expected to be greater in clear as opposed to stained waters, but the difference in benthic contribution between the two water types is much greater in shallow than deep lakes (Fig. 3).

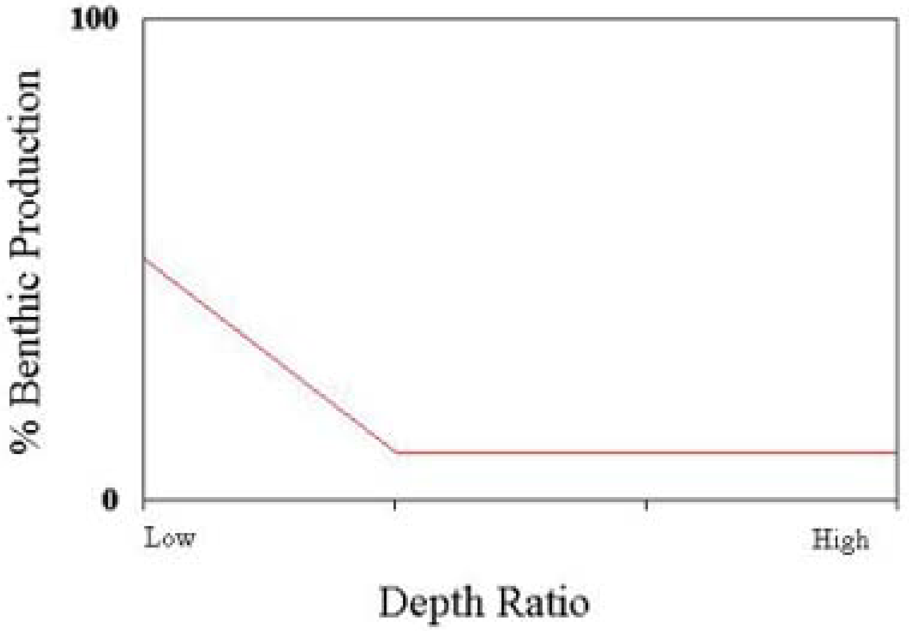

**FIGURE 2.** Conceptual relationship between lake morphometry (depth ratio, defined as mean depth/maximum depth) and the relative contribution of benthic primary production. See text for additional details.

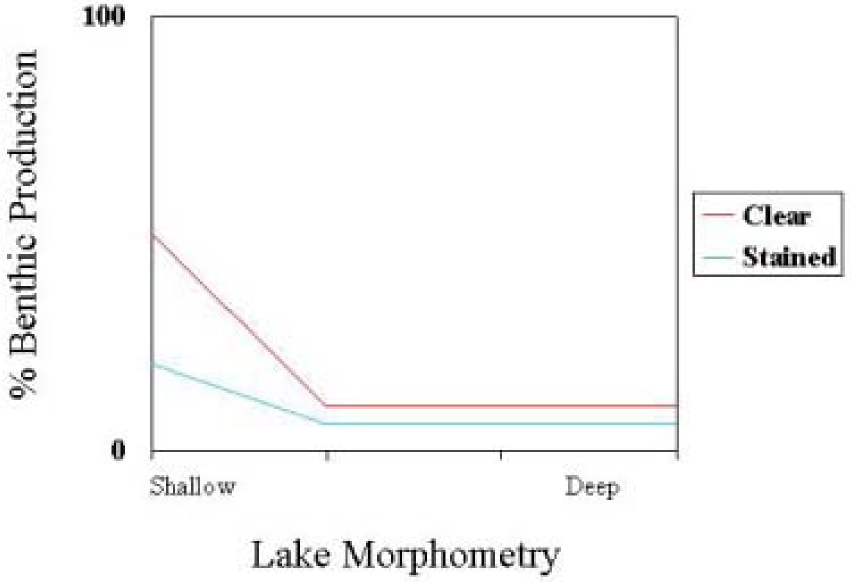

**FIGURE 3.** Conceptual relationship between lake morphometry and the relative contribution of benthic primary production. Note that the influence of lake morphometry is damped when water color is stained. See text for additional details.

Another informative morphometric parameter is depth ratio (mean depth/maximum depth), which is an index of the steepness of the sides of the lake[14,15]. Lakes with a low depth ratio often have extensive shallow areas that can support benthic algae. Lake Thingvallavatn has a depth ratio of 0.28, which partly explains the high contribution of benthic primary production[29]. Similarly, Castle Lake, California (mean depth 11.4 m) has a depth ratio of 0.3 and extensive shallow areas. The littoral plane in Castle Lake supports a high biomass of epipelon that accounts for as much as half the total primary production in the lake[30].

If we assume that the relative contribution of benthic primary production is an index of its relative importance in ecosystem function[31], then lake morphometry will tell us a great deal about the relative contribution of benthic algae to lake metabolism. According to our conceptual model, we anticipate that the maximum contribution of benthic algae to total lake metabolism will be inversely related to depth. The contribution of periphyton to whole lake primary production in shallow lakes varied between 1 and 99% along a eutrophication gradient[26]. It is probable that individual shallow lakes switch between phytoplankton and periphyton dominance similarly to the way in which they shift between phytoplankton and macrophyte dominance[32] because light availability to the sediments can be reduced markedly by increases in phytoplankton or suspended solids. For instance, Lake Okeechobee is a very large, shallow (1,800 $km^2$, mean depth 2.7 m) subtropical lake located in Florida with a depth ratio of approximately 0.5. The biomass contribution by attached algae varies dramatically by season; in winter, attached algae accounted for only 1% of the total chlorophyll *a*, but in summer this percentage increased to 39%[33]. Morphometry explains some large-scale, among-lake differences in the relative contribution of benthic algae to lake metabolism. However, the importance and function of periphyton is influenced also by the distribution and abundance of different substrata in the littoral zone and by biotic interactions.

## Substrate Composition

Although substrate does not affect periphyton metabolism directly, it does have the potential to influence metabolism indirectly, largely through nutrient release. Benthic periphyton communities in lakes have the potential to obtain their nutrients from the water column, the substrate to which they are attached, or from internal cycling within the periphytic mat[34,35]. The trophic status of the water body, the substrate type, and the thickness of the mat all influence nutrient kinetics and the relative importance of the water column or substrate as the nutrient source[36].

It has been theorized that the importance of the water column in supplying nutrients to periphyton is greater in eutrophic lakes[37,38]. This makes energetic sense because the cells can use the abundant inorganic nutrients in the water column, and would not need to process organic forms of nutrients. The results from a study in Lake Okeechobee were consistent with this idea. Under turbid and elevated phosphorus conditions, periphyton relied mostly on inorganic phosphorus but under clear-water, nutrient-poor conditions, dissolved organic phosphorus (DOP) became a more important source of phosphorus[39]. In addition, studies from oligotrophic and mesotrophic lakes have shown that epiphytic communities growing on artificial substrates that cannot serve as a source of phosphorus, rely more heavily on organic phosphorus for their P needs than epiphyton growing on natural macrophytes[40,41].

Periphyton grows on many different substrate types in lakes, including mud sediments (epipelon), sand grains (epipsammon), rocks (epilithon), macrophytes (epiphyton), and wood (epixylon). Some of these substrate types have the potential to release nutrients to the attached periphyton and influence periphyton metabolism, although as noted above, the lake's trophic status may influence the role of substratum-derived nutrients. Hansson[42,43] demonstrated that epipelon can significantly reduce nutrient availability in the water column due to uptake of diffusing nutrients, and that this affect was most distinct in a nutrient-poor lake. Similarly, Hagerthey and Kerfoot[44] demonstrated that inflowing groundwater was a significant source of nutrients for epipsammon in oligotrophic Sparkling Lake, Wisconsin. The capacity of substratum to influence periphyton productivity was demonstrated in laboratory experiments in which productivity of natural periphyton assemblages on wood was only 10% of productivity on sediments. Furthermore, epixylon but not epipelon responded positively to water-column fertilization[45]. Thus, unconsolidated sediments are often significant sources of nutrients for associated periphyton[42,44,46], but hard surfaces such as wood and rocks are not[38,45], and the importance of macrophytes as a nutrient source depends on the trophic status of the lake[41].

The influence of substrata as potential sources of nutrients is confounded by the nonrandom distribution of substrata with respect to depth, and consequently, light. For instance, wave action in well-lit, near-shore areas removes organic matter, leaving behind rocks or sand. Sand and rocks are nutrient-poor habitats that are associated with low rates of periphyton production (but see Romaní and Sabater[47]), and waves may disrupt algal mats or prevent mat development altogether[48,49]. Conversely, organic sediments accumulate in deeper areas of the lake. In these areas, disturbance is low, and pore-nutrient concentrations can be high[45], both of which tend to lead to well-developed algal mats and high rates of production. Thus, areas of maximum light, maximum nutrient availability, and minimum disturbance rarely coincide at a single depth. Instead, maximum periphyton production may occur at some intermediate depth where none of these critical parameters is optimal.

## Periphyton-Phytoplankton Interactions

Periphyton and phytoplankton grow in different, but adjacent, habitats. Both types of algae require nutrients and light, but these resources are not equally available in the two habitats. Resources move across habitat boundaries making exploitative competition likely. Conceptual models[18,19,20] postulate that periphyton will dominate in oligotrophic lakes because epipelic algae have access to sediment nutrients. In oligotrophic lakes, low phytoplankton densities allow enough light to reach the sediments for periphyton to maintain high production rates, but in eutrophic lakes, phytoplankton biomass may impose light limitation on benthic algae. The models[18,20] predict that areal periphyton production peaks at mid-TP levels when both nutrient and light are abundant. We used the literature data from Table 1 to test whether this hypothesized pattern could be detected in the few lakes for which whole-lake production estimates are available. Many of the studies did not have TP measurements so we used phytoplankton production as an index of a eutrophication gradient (Fig. 4). In oligotrophic lakes, both periphyton and phytoplankton production are low, though periphyton production often exceeds phytoplankton production at the whole-lake scale. At the opposite extreme, when phytoplankton production is very high, periphyton production is invariably low. At intermediate levels of phytoplankton production, the contribution of periphyton to whole-lake production is highly variable. This variation likely stems from site-specific differences in grazing pressure, habitat suitability, localized disturbance, and pelagic trophic structure. However, the distribution

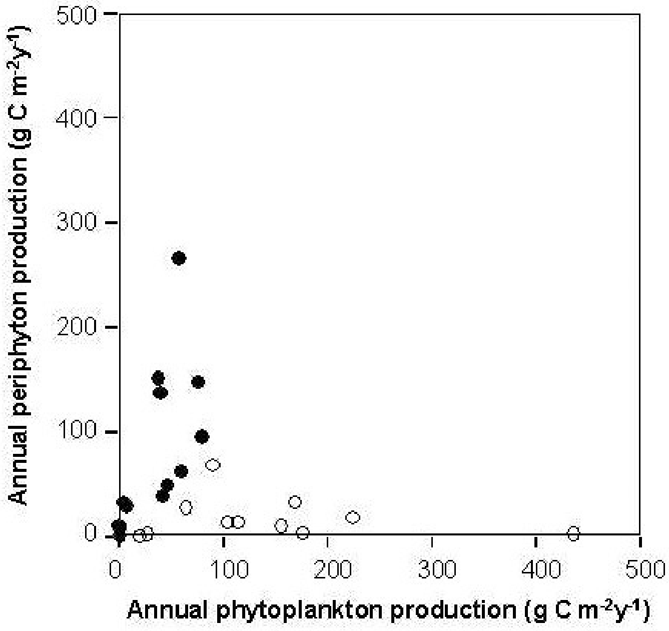

**FIGURE 4.** Areal whole-lake periphyton productivity is a unimodal function of phytoplankton productivity. Data are from Table 1. Only lakes for which periphyton productivity was measured on all substrata were included (Lakes 239 and 240 were omitted). Closed symbols are lakes for which periphyton productivity ≥ phytoplankton productivity.

supports the patterns expected if interactions for light and nutrients strongly affect the relative contributions of periphyton and phytoplankton to whole-lake primary production. The few field studies that have experimentally tested this inverse relationship at localized scales have also confirmed its existence[7,42,50,51,52]. In the next sections we explore the mechanisms underlying competition between phytoplankton and periphyton.

## Competition for Nutrients

Nutrient cycling in lakes often ignores the benthic primary producers, focusing instead on the phytoplankton, bacteria, and perhaps zooplankton compartments. Sedimentation to the benthos is usually simply a loss term. Here we consider the water column, the sediments, and benthic and pelagic primary producers as compartments among which nutrients are transferred in both directions. For simplicity, we do not address the role of consumers, though they are an important and dynamic compartment for nutrients. Interactions between periphyton and phytoplankton for nutrients have been explored at several different scales, often with very different conclusions about the relative importance of periphyton and phytoplankton in lake nutrient cycles and different predictions regarding the outcome of competition between the two groups.

One way of assessing the competitive abilities of phytoplankton vs. periphyton is to compare their nutrient kinetics. Most evidence supports the hypothesis that phytoplankton have higher nutrient uptake rates from the water column than periphyton. In nitrogen deficient Californian lakes (Lake Tahoe and Castle Lake), uptake rates of nitrogen were fitted to Michaelis-Menten dynamics[53,54]. In both lakes, half saturation constants ($K_m$) for phytoplankton were similar to *in situ* nitrogen concentrations, indicating a high affinity for dissolved nitrogen. In contrast, $K_m$ values for periphyton were up to 100 times those of phytoplankton. Such affinities indicate that phytoplankton can out-compete periphyton for water-column nitrogen. Similar patterns were observed for uptake of water-column phosphorus in Lake Okeechobee. Specific uptake rates of phosphorus for phytoplankton were 10 to 20 times those of either epiphytes or epipelon[52]. These results were consistent with the findings of Steinman et al.[55], who also found that phytoplankton in Lake Okeechobee had higher phosphorus uptake rates and a lower $K_m$ for phytoplankton than benthic charophytes. Literature data supported the pattern of phytoplankton having a competitive advantage over periphyton for water-column nutrients, as indicated by the generally higher half saturation constants for periphyton than for phytoplankton[52].

The experiments on nutrient uptake rates from the water column suggest that phytoplankton are superior competitors for nutrients. However, this conclusion is based on short-term bottle incubations using isolated communities. Such experiments may not reflect longer-term, steady-state conditions in heterogeneous environments because retention and recycling of nutrients may differ among communities. For instance, when phosphorus uptake by phytoplankton and epipelon was measured simultaneously within sediment-phytoplankton microcosms, epipelic algae were responsible for uptake of 70 to 95% of added phosphorus in experimental cores[56]. In mesocosm studies, the majority of added phosphorus is often sequestered by the periphyton on mesocosm walls, although one study showed that only a small fraction of total phosphorus is incorporated by periphyton[51,57,58]. Although this experimental artifact impedes extrapolation from the mesocosm to the whole lake, it also emphasizes an important point: even if phytoplankton exhibit more rapid uptake of water-column nutrients, periphyton appears to be more effective at nutrient retention. For instance, phytoplankton were much more efficient than epipelon in sequestering water-column nutrients in small-scale, short-term experiments in Castle Lake. This led to the conclusion that epipelic algae were unimportant in uptake of water-column nutrients[30]. However, when mesocosm and whole-lake fertilization experiments were conducted in this lake, epipelon took up 56% of added nitrogen. This was not anticipated based on small-scale uptake results and suggests that: (1) small-scale studies did not accurately represent the natural system, perhaps due to under-representation of periphyton or over-representation of phytoplankton; (2) periphyton retention of nutrients is much higher than for phytoplankton; and/or (3) phytoplankton loss rates, such as from sinking or herbivory, were not accurately portrayed in the bottle experiments.

An additional consideration is the depth at which nutrients enter into a lake system. Nutrients may enter in the metalimnion layer if the inflowing water is colder (and thus, denser) than the water in the epilimnion layer[59,60]. Wurtsbaugh et al.[61] used limnocorrals to artificially enrich epilimnetic vs. metalimnetic layers of an oligotrophic, alpine lake. They found that periphyton chlorophyll never accounted for >4% of total chlorophyll in the control treatment, but composed 20 to 50% of total chlorophyll in the epilimnion enrichment treatment and 1 to 20% of total chlorophyll in the metalimnion enrichment treatment. They concluded that periphyton communities may assimilate a significant fraction of nutrients in an epilimnetic fertilization (rather than the phytoplankton), and thus divert nutrients from the pelagic food web. This conclusion presumes that periphyton communities are trophic dead ends, which is questionable (see below).

A second problem with focusing on phytoplankton-phytoplankton competition for water-column nutrients is that the water column is not the only, and perhaps often not even the most important, nutrient pool in lakes. Often the sediments constitute the largest pool of nutrients, and sediments in contact with the epilimnion may be a substantial source of phosphorus to phytoplankton during the stratified summer season[62]. Nutrient concentrations in sediment pore water can be 5 to 100 times higher than water-column concentrations[44,45,63]. Algae growing on sediments can oxygenate the sediment surface and reduce transport of sediment nutrients to the overlying water[64]. A series of experiments demonstrated that epipelic algae use sediment nutrients for growth and in doing so reduce the quantity of nutrients available to phytoplankton[42,43]. This active uptake of nutrients resulted in lower phytoplankton biomass and production in the water column in both laboratory aquaria and mesocosm experiments. The importance of periphyton uptake across the sediment-water interface will likely have the greatest effect on phytoplankton in oligotrophic or shallow lakes. In oligotrophic Sparkling Lake, epipsammic algae had higher growth rates and biomass in zones of inflowing, high-nutrient groundwater compared to downwelling zones[44]. Periphyton intercepted these nutrients, rendering them unavailable to phytoplankton. These sediment nutrient studies allow us to view the nutrient kinetic experiments in a different light. The low affinity of epipelon for water-column nutrients may simply reflect the high availability of nutrients in the sediment pore water. Of the three groups of periphyton studied in Castle Lake, epipelic algae showed the lowest affinity for water-column nitrogen while filamentous green algae in the splash zone of the shallow littoral showed the most similarity to phytoplankton uptake rates. Epipelon have direct access to sediment nutrients, whereas filamentous algae do not grow as a cohesive mat, and are dependent on the water column for nutrients.

As the preceding discussion demonstrates, the spatial segregation of phytoplankton and periphyton into different habitats does not preclude competitive interactions between benthic and pelagic primary producers. However, the degree of habitat overlap can affect the strength of these interactions. Again, lake morphometry will determine how these resources are allocated between primary producers; the greater the overlap between littoral and pelagic habitats, the more likely it is that strong interactions for resources will occur. In a large, deep lake the majority of epilimnetic water will overlie the hypolimnion and seasonal exchange of nutrients with hypolimnetic waters or eddy diffusion, rather than nutrient exchange with littoral sediments, will have a strong influence on nutrient availability to phytoplankton. But in shallow lakes phytoplankton production may occur predominantly over littoral sediments and both periphyton sequestration of nutrients and light attenuation by phytoplankton will strongly affect periphyton productivity patterns.

It is anticipated that the relationship of light and nutrients on periphyton may be complex and may vary on a site-specific basis because of the confounding influences of temperature, substrate, and herbivory. Experimental studies have shown that the influence of nutrient supply on periphyton is reduced under low light regimes[65], presumably because lower photosynthetic rates result in reduced demand for nutrients[66]. In addition to the potential interactive effects of light with nutrients, competition for light alone can influence periphyton dynamics in lakes, as discussed in the next section.

## Competition for Light

Irradiance is a critical resource for periphyton and has been shown to influence periphyton community structure[67], biomass[67,68], and metabolism[69]. The role of irradiance can be addressed at several different spatial scales: (1) at the landscape/watershed scale, where characteristics of the drainage basin affect the nutrient loads and the amount of DOM entering the receiving water body; (2) at the scale of the lake, where phytoplankton and periphyton may compete for light; and (3) within the periphyton matrix, where light attenuation and absorption occur at a fine scale.

At the coarsest spatial scale, drainage basin characteristics can strongly influence lake dynamics. Nutrient loading associated with land use can affect lake structure and function[70,71,72]. Nutrients from agriculture (e.g., commercial fertilizers, biosolids, and animal manure) are rich in nitrogen and phosphorus, elements that often limit primary production in aquatic ecosystems. Additions of these nutrients above a lake's natural assimilative capacity can result in ecological impairment, such as algal blooms and changes in food web interactions[71,73,74]. Phytoplankton blooms will result in less light being transmitted to the benthos, influencing periphyton growth (see below).

In addition, watersheds that drain highly organic soils can result in colored water, rich in DOM. Light can be a limiting resource for benthic algae in such environments because of reduced light penetration through the water column. Related to the role of DOM is ultraviolet radiation, which can have both direct and indirect affects on periphyton structure and function. Most work on UVR in lakes has been focused on planktonic communities[75,76], but periphyton is also at risk to UVR, especially in oligotrophic systems. Although epipelic species may migrate downward into sediments during the daylight to avoid UVR exposure[77], Higley et al.[78] reported that *Anabaena circinalis*, the dominant periphyton species in Castle Lake was sensitive to UVR, possibly because its nitrogen-fixing capacity was inhibited by UVR. The major indirect effect of UVR is via photolysis of DOM[79]. Sunlight has the potential to photobleach the organic matter, which may increase light penetration through the water column[80] and result in more labile forms of carbon for bacterial or algal uptake[81]. This can have either positive or negative affects on periphyton: positive, if anoxygenic bacterioplankton are stimulated by photochemical transformation of DOM and they outcompete phytoplankton for nutrients, thereby resulting in less light being absorbed in the water column; negative, if phytoplankton respond positively to the transformed DOM, thereby resulting in less light reaching the benthos.

At the intermediate scale of individual lakes, phytoplankton should have a competitive advantage over periphyton for light because phytoplankton circulate in the water column and can intercept light before it reaches periphyton. Although numerous studies have addressed the role of light in lakes, little attention has been paid to explicitly examining how periphyton and phytoplankton compete directly for light. Observational studies have shown that an inverse relationship often exists between periphyton or benthic vegetation and phytoplankton[82,83]. Experimental fertilization of lakes led to a decline in whole-lake periphyton primary production because phytoplankton reduced light availability for periphyton[21]. Havens et al.[50] suggested that in shallow zones, resuspended meroplankton from the benthos may be an important source for phytoplankton biomass, providing an intimate linkage between the two communities[84].

A more direct approach to assess competitive ability of phytoplankton vs. periphyton is to compare the photosynthetic parameters of the communities, as revealed by photosynthesis-irradiance (P-I) relationships. Surprisingly, only a few P-I studies have been performed with lake periphyton, perhaps because of logistical problems associated with *in situ* manipulations, and even fewer studies have compared P-I curves of periphyton vs. phytoplankton. Hill[68] reviewed the P-I literature for lotic periphyton and reported that photosynthetic saturation ($I_k$) usually occurred between 100 and 400 $\mu$mol $m^{-2}$ $s^{-1}$, although this value could be substantially lower or higher, depending on whether periphyton cells were growing in low or high-light environments, respectively[17]. Turner et al.[85] reported periphyton photosynthesis in an oligotrophic lake to be saturated at an irradiance of approximately 200 $\mu$mol $m^{-2}$ $s^{-1}$, well within the range reported by Hill[68] for lotic periphyton. Steinman et al.[55] found $I_k$ values of 145 $\mu$mol $m^{-2}$ $s^{-1}$ for charophytes in a turbid, shallow lake, whereas Howard-

Williams et al.[86] reported $I_k$ values approximately half that level for charophytes in an oligotrophic lake. Maximum photosynthetic rates and $I_k$ values were inversely related to the depth from which periphyton was collected in soft water lakes in Michigan, and the range of potential primary productivity of the epipelon was tightly coupled to the *in situ* light range[45]. This indicates that P-I parameters for lake periphyton vary according to antecedent environmental conditions, in a manner similar to stream[68] and estuarine systems[87].

The finest scale at which to consider the impact of light on lake periphyton is attenuation through the matrix[8]. The consortium of organic and inorganic matter results in a rapid decline in light transmission through the periphyton mat, with photic zones often restricted to the upper centimeter or less[88,89,90]. Selective absorption of light also influences the quality of the photosynthetically available radiation that penetrates through the mat; marine studies with microphytobenthos have shown that blue and red wavelengths are selectively reduced due to absorption by chlorophyll *a*[91].

The periphyton matrix can have other impacts besides light attenuation. An exhaustive literature review evaluating photosynthetic rates in phytoplankton, macrophytes, and attached microalgal mats found that maximum chlorophyll-specific photosynthesis was substantially lower in the attached algal mats than the other two communities[92]. Although methodological differences may account for a portion of the reduced photosynthesis, the authors conclude that diffusion limitation of inorganic carbon and dissolved oxygen was the most plausible explanation.

## Periphyton in Lake Food Webs

One effect of the predominant interest in phytoplankton production in limnology has been a general lack of appreciation of periphyton as a resource for consumers in lakes[8]. There are parallels with trends in research into river food webs; early stream studies showed that terrestrial inputs of detritus dominated carbon budgets and, inferred, fuel ecosystem productivity[93]. Eventually it was understood that periphyton may form a major resource base for invertebrates in flowing water ecosystems. Even when biomass is low, periphyton communities can be a critical food resource because of their high turnover rates[94]. This realization produced a rich array of studies detailing the energetic importance of periphyton in streams[95]. In lakes, productivity of phytoplankton is usually assumed to form the autotrophic base of ecosystem productivity of higher trophic levels. Obviously, phytoplankton-fixed carbon is important to zooplankton and profundal zoobenthos, but littoral zoobenthos are a key component of many fish diets[97,98] and have direct access to periphyton. Although little attention has been paid to relationships between benthic primary and secondary productivity, it is reasonable to expect that the contribution can be high given the substantial contribution of periphyton to whole-lake primary productivity in many lakes (Table 1).

There are many potential pathways for periphyton to be incorporated into lake food webs. Conspicuous benthic invertebrates that graze periphyton include snails, caddisflies, crayfish, chironomids, and mayflies. Nor are benthic invertebrates the only group that grazes periphyton. There are many zooplankton that associate with macrophytes in the littoral zone and it is likely that these graze epiphytes[99]. Filamentous algae are an important diet item of many minnows (*Cyprinidae*)[100]. In African rift valley lakes some taxa in the diverse assemblage of cichlids specialize on grazing periphyton and divide up habitat to the extent that they graze on different surfaces or at different depths[101,102].

Grazers can exert strong top down effects on periphyton. Although most periphyton-grazer studies are conducted in streams[95], much attention has been paid to snail-periphyton-macrophyte interactions in lakes. Heavy epiphyte burdens can lower light availability to macrophytes. Snails substantially reduce epiphyte biomass, thereby potentially benefiting the macrophytes[103,104,105]. Caddisflies are another conspicuous grazer in lakes that reduce periphyton abundance and production on rocks, wood, and sediments[106; Vadeboncoeur, unpublished data]. Several studies have demonstrated that profundal chironomid production is correlated with the availability of phytoplankton detritus[107,108]. However, the generally higher densities of chironomids in the littoral zone compared with the profundal zone[109] suggests that periphyton can be an important resource for chironomids. Chironomids often constitute the majority of benthic invertebrate biomass and production, and several studies show that littoral chironomid production can be dependent on periphyton production. When nutrients were added to mesocosms, periphyton accumulation and chironomid densities on mesocosm walls were positively related to nutrient addition rates, indicating that the chironomids likely were food limited on the newly colonized substrate[51]. Furthermore, chironomids exerted a strong top down suppression of periphyton on walls in another mesocosm study[57]. Grazing studies and gut content analysis show that periphyton is an important resource for many other benthic taxa as well[73,100]. Strong suppression of benthic algae by grazers may, by inference, lead to the conclusion that periphyton is an important resource in lake food webs. However, caution should be exercised because strong top down control by animals on a preferred resource does not necessarily mean that the resource is important if the food item is rare in the environment. Given the ubiquity of periphyton on submersed surfaces, this is probably not the case. However, the ability of grazers to exert a strong top down effect does not in any way elucidate the importance of those grazers to organisms higher in the food web.

Connectivity food webs are important tools for elucidating the role of periphyton in energetic pathways in lakes. Havens et al.[73] developed food web models for the pelagic (218 taxa) and littoral (324 taxa) zones of Lake Okeechobee and found that the resource most utilized by consumers was the periphyton/detritus compartment (the analysis did not distinguish between the two). This finding is consistent with results from other lakes[111] and streams[112]. Such donor-controlled detrital resources can help sustain high rates of secondary productivity because detritivores do not affect the delivery rate of detritus. Such food webs are especially powerful when used in conjunction with stable isotope analysis, which gives an index of the relative energy flow among different compartments[113].

Connectivity food webs are descriptors of the trophic relationships among organisms but do not provide information on the relative importance of phytoplankton vs. periphyton to higher trophic levels. Ratios of $^{13}C$:$^{12}C$ ($\delta^{13}C$) provide a tracer for different types of primary producers through food webs. The DIC sequestered by phytoplankton during photosynthesis often originates from carbon respired from the watershed or sediments in oligotrophic lakes, while in eutrophic lakes, high carbon demand from phytoplankton causes influx of atmospheric carbon[114]. In either case, phytoplankton discriminate strongly against $^{13}C$ and typically have a $\delta^{13}C$ ratio between -24 and -38‰[115]. In contrast, benthic algae take up DIC across a boundary layer that surrounds the algal mat. This causes a slight carbon deprivation that leads to less discrimination by benthic algae compared to phytoplankton. Therefore, benthic algae have a less negative signal than phytoplankton, typically ranging between -16 and -30‰. These differences in carbon signals are maintained throughout the food web and are reflected in the isotopic signals of all subsequent consumers. Thus, $\delta^{13}C$ provides a natural tracer of the autotrophic basis of secondary production in lake food webs.

There are several methodological considerations when using stable isotopes to describe food webs. One of the strengths of stable isotope analysis is that it gives a composite measure of an organism's feeding habits integrated over time. However, phytoplankton and periphyton have short turnover times and temporally variable isotopic signatures relative to the organisms that they support in the food web[116,117,118]. Thus, a point measurement of the isotopic signal of primary producers may not represent the composite signal that primary consumers experience during growth. This limitation can be overcome by using a primary consumer with known feeding habits as a time-integrated index of the primary producer signal. For instance, mussels are a group of long-lived filter feeders that are used extensively as a baseline signal for phytoplankton $^{13}C$ and $^{15}N$[119] ($^{15}N$ is used to determine trophic position of consumers). Currently there is no standard primary consumer of periphyton analogous to mussels for phytoplankton, but some species of snails with known feeding behaviors might be appropriate. Using a primary consumer, rather than a primary producer, is also advisable for establishing a $\delta^{15}N$ baseline because fractionation of $^{15}N$ between plants and herbivores is much more variable (i.e., not consistently 3.4‰) than for subsequent trophic levels[120,121].

The $\delta^{13}C$ and $\delta^{15}N$ signals of periphyton in lakes are also highly variable spatially[116]. Substrata and depth gradients both contribute to this variability. For instance epipelon tends to have more negative $\delta^{13}C$ signals than epilithon and epiphyton, and this pattern is consistent across numerous lakes (Vadeboncoeur, unpublished data). Epipelon $\delta^{13}C$ decreased with depth in humic softwater lakes (Vadeboncoeur, unpublished data), and literature review showed that $\delta^{13}C$ in benthic primary consumers consistently decrease with depth[121]. Such spatial and temporal variation in isotopic signals impose limitations on the quantitative conclusions made from food webs based on stable isotopes alone because it can be difficult to determine exact benthic end points for isotopic mixing models.

Complications in determining baseline isotopic signals of primary producers also arise because the algae most apparent to the observer are not necessarily the periphyton that is incorporated into the food web. Grazers can crop diatom mats on rocks or macrophytes to a thin layer, but grazers rarely consume large filamentous green algae of the Zygnematales order (e.g., *Spirogyra*, *Mougeotia*, and *Zygnema*). Filamentous green algae form clouds of metaphyton that are highly conspicuous, but unimportant in the food web[122]. In contrast, heavily grazed, and therefore inconspicuous, diatom mats can be both the most relevant and most difficult periphyton to collect. Epipelic algae are often highly productive but are cryptic and not easily separated from the sediments on which they grow. Nevertheless, stable isotope analysis indicates that epipelon is an important resource for grazers (Vadeboncoeur unpublished data). Perhaps the presence of conspicuous, dense algal mats should alert researchers to the possibility that a particular category of periphyton is an unimportant food resource.

Despite these potential problems, stable isotope analysis has contributed greatly to our increased understanding of the role of periphyton in lake food webs. Early studies allowed researchers to determine the relative contributions of terrestrial detritus, phytoplankton, and periphyton to benthic invertebrate diets[123]. Although spatial and temporal variability frustrated efforts to calculate the exact magnitude of the contribution of benthic algae to energy flow in Australian billabongs[124], the study revealed that snails and Leptocerid caddisflies consumed primarily periphyton while zooplankton, mussels, and chironomids consumed primarily phytoplankton, and crayfish consumed macrophyte detritus. Determining the importance of periphyton to invertebrates is interesting, but the real power of stable isotope analysis is to track the importance of various primary producers through the food web, ultimately to top predators. Hecky and Hesslein[125] used a qualitative and synthetic approach to determine if fish in tropical, temperate, and arctic lakes were ultimately reliant on carbon fixed by phytoplankton or periphyton. Top predators generally had $\delta^{13}C$ signatures midway between benthic and pelagic primary producers indicating that periphyton and phytoplankton were of similar importance in food webs. This concurs with diet analysis that shows that 15 North American fish taxa derive, on average, 65% of their food from zoobenthos[97]. In very turbid, light limited lakes, a periphyton signal was difficult to detect even in benthic primary consumers[125]. This is not surprising given the decreasing contribution of periphyton to whole lake primary production associated with increased TP[21].

Although few studies have intentionally explored the energetic importance of benthic algae, many subsequent stable isotope studies of fish support the conclusion[25] that periphyton is a major component of lake food webs. Arctic char in Char Lake, Canada are heavily dependent on carbon fixed by filamentous periphyton[126], which together with benthic mosses constitute about 80% of whole-lake primary production[127]. In lakes with diverse fish assemblages, some species show strong use of benthic algal carbon while other species appear to be dependent on phytoplankton carbon. In subtropical Lake Okeechobee, fish in marshy areas had far more variable $^{13}C$ signals then those occupying open water habitat because marsh fish have access to periphyton-, macrophyte-, and phytoplankton-derived carbon[128]. It is intuitively logical that there are strong energy flows from periphyton to fish assemblages in small shallow lakes[129,130]. However, even in a large Alaskan lake (Iliamna Lake, 2,622 $km^2$, mean depth 44 m, maximum depth 393 m), the substantial use of littoral carbon by diverse taxa is impressive. Dolly Varden (*Salvelinus malma*), Rainbow Trout (*Oncorhynchus mykiss*), and Sockeye Salmon (*Oncorhynchus nerka*) obtained 46, 25, and 10% of their carbon from littoral sources, respectively, and sculpin (*Cottus aleuticus*) had an almost entirely benthic diet[131]. Fish in other deep lakes also rely on carbon fixed by littoral algae. In Bow Lake, Canada (mean depth 22.9 m, maximum depth 51 m), mountain whitefish exploited only littoral carbon while lake trout exploited both pelagic and littoral carbon, exhibiting an increasingly littoral signal with increasing size[132]. Stable isotope analysis demonstrated that high rates of periphyton productivity support the diverse and productive littoral zone fish assemblage in Lake Malawi (28,800 km$^2$, maximum depth 485 m) in the African rift valley[102,125,133]. This complemented diet analysis, which showed that some cichlids in Lake Malawi directly consume diatoms and the cyanobacterium, *Calothrix*[101].

The above studies indicate that in lakes with productive littoral zones, there are fish that exploit benthic primary production. No littoral signal was detected from pelagic fish in Lake Superior[134], but fisheries in the bays and wetlands of the Laurentian Great Lakes were dependent on benthic carbon and had higher yields than in pelagic fisheries[135]. The littoral food web of Lake Baikal, the deepest lake in the world, showed a strong reliance on benthic algae to a depth of 50 m[136]. These studies challenge the common generalization periphyton is an important component of food webs only in relatively small, shallow lakes. Rather, there is some evidence that periphyton is largely unimportant in the food webs of very eutrophic or turbid lakes[26,125]. Many of the studies cited above were designed to trace fish diets rather than to quantify the trophic significance of periphyton. More thorough studies are needed to develop littoral food webs that lead to fish. James et al.[110] used a compelling combination of stable isotopes and diet analysis to show that the major energetic pathway in an oligotrophic New Zealand lake (maximum depth = 200 m) flowed from epiphytes to trichopterans and chironomids to fish. Similar robust food webs need to be developed for other lakes.

## SUMMARY

Periphyton communities have received relatively little attention in the study of lentic systems. We have argued that attached algae often play a critical role in the nutrient cycles, energy flow, and food webs of lakes. The degree to which these communities influence lake function can be conceptualized in a spatial hierarchy, where at the landscape scale, lake morphometry determines the relative availability of habitat for benthic and pelagic primary producers[3]. Watershed-related properties, such as DOM loading, indirectly influence periphyton by altering light attenuation in lakes. Within the lake, habitat availability and heterogeneity influence the abundance and productivity of the periphyton. At a finer spatial scale, periphyton and phytoplankton compete for resources, such as light and nutrients.

Although lake nutrient cycles traditionally treat the lake bottom as a physical nutrient sink, periphyton communities are an active biotic component of nutrient transfer between the water column and benthic surfaces[44,46,64]. Phytoplankton have higher nutrient uptake rates from the water column than do periphyton, but periphyton appears to be better at retaining nutrients once they are sequestered[30,54,56]. Furthermore, periphyton can regulate the flux of nutrients from unconsolidated sediments to phytoplankton. Periphyton communities are important in nutrient cycles of oligotrophic lakes, but with increasing water-column TP, the ability of periphyton to sequester benthic nutrients diminishes in importance relative to the ability of phytoplankton to intercept light before it reaches benthic surfaces. Thus, there is a unimodal relationship between periphyton and phytoplankton production (Fig. 4).

Phytoplankton are generally treated as the trophic basis of production in lakes, but grazing studies, gut content analysis, and stable isotope analysis all indicate that periphyton fuels food webs in many lakes[26,73,125]. Many species of fish exploit benthic invertebrates extensively but the energetic link between periphyton and zoobenthos is poorly researched[97]. Morphometry alone can be a misleading guide to the role of periphyton in lake food webs. Attached algae are important in food webs in very large oligotrophic lakes[110,136], while initial evidence indicates periphyton is important in small clear lakes but plays a lesser role in shallow lakes that are extremely eutrophic[26].

Stable isotope analysis is providing compelling evidence that littoral zones are not just a source of shelter and habitat for fish; the littoral zone provides a critical foraging base, ultimately derived from periphyton.

Models of lake ecosystem function tend to focus on water-column and phytoplankton dynamics, downplaying the importance of the benthic layer and periphyton, in particular. Increasing attention is being paid to benthic-pelagic interactions[7,51,137], as new techniques have provided evidence that periphyton communities can play important roles in lakes. Formal recognition of the functional importance of periphyton in lakes, either via empirical studies or in model development and validation, will provide a more complete understanding of lake structure and function.

## ACKNOWLEDGEMENTS

Yvonne Vadeboncoeur was supported by a postdoctoral fellowship from Groupe de recherche interuniversitaire en limnologie et en environnement aquatique. Alan Steinman acknowledges the patience and scientific insights of Karl Havens, without whom this review would not have been written. Comments from an anonymous reviewer improved the manuscript.
